# Gold Nanoparticles Induce Oxidative Stress and Apoptosis in Human Kidney Cells

**DOI:** 10.3390/nano10050995

**Published:** 2020-05-22

**Authors:** Maria Enea, Eulália Pereira, Miguel Peixoto de Almeida, Ana Margarida Araújo, Maria de Lourdes Bastos, Helena Carmo

**Affiliations:** 1 UCIBIO/REQUIMTE, Laboratório de Toxicologia, Departamento de Ciências Biológicas, Faculdade de Farmácia, Universidade do Porto, Rua Jorge Viterbo Ferreira, 228, 4050-313 Porto, Portugal; ana.margarida.c.araujo@gmail.com (A.M.A.); mlbastos@ff.up.pt (M.d.L.B.); 2 LAQV/REQUIMTE, Departamento de Química e Bioquímica, Faculdade de Ciências, Universidade do Porto, Rua Campo Alegre, 687, 4169-007 Porto, Portugal; mpda@fc.up.pt

**Keywords:** gold nanoparticles (AuNPs), HK-2 cells, nanospheres, nanostars, size, capping

## Abstract

Gold nanoparticles (AuNPs) are highly attractive for biomedical applications. Therefore, several in vitro and in vivo studies have addressed their safety evaluation. Nevertheless, there is a lack of knowledge regarding their potential detrimental effect on human kidney. To evaluate this effect, AuNPs with different sizes (13 nm and 60 nm), shapes (spheres and stars), and coated with 11-mercaptoundecanoic acid (MUA) or with sodium citrate, were synthesized, characterized, and their toxicological effects evaluated 24 h after incubation with a proximal tubular cell line derived from normal human kidney (HK-2). After exposure, viability was assessed by the MTT assay. Changes in lysosomal integrity, mitochondrial membrane potential (Δ*Ψ*m), reactive species (ROS/RNS), intracellular glutathione (total GSH), and ATP were also evaluated. Apoptosis was investigated through the evaluation of the activity of caspases 3, 8 and 9. Overall, the tested AuNPs targeted mainly the mitochondria in a concentration-dependent manner. The lysosomal integrity was also affected but to a lower extent. The smaller 13 nm nanospheres (both citrate- and MUA-coated) proved to be the most toxic among all types of AuNPs, increasing ROS production and decreasing mitochondrial membrane potential (*p* ≤ 0.01). For the MUA-coated 13 nm nanospheres, these effects were associated also to increased levels of total glutathione (*p* ≤ 0.01) and enhanced ATP production (*p* ≤ 0.05). Programmed cell death was detected through the activation of both extrinsic and intrinsic pathways (caspase 8 and 9) (*p ≤* 0.05). We found that the larger 60 nm AuNPs, both nanospheres and nanostars, are apparently less toxic than their smaller counter parts. Considering the results herein presented, it should be taken into consideration that even if renal clearance of the AuNPs is desirable, since it would prevent accumulation and detrimental effects in other organs, a possible intracellular accumulation of AuNPs in kidneys can induce cell damage and later compromise kidney function.

## 1. Introduction

Gold nanoparticles (AuNPs) present a wide range of biomedical applications firstly due to their plasmonic properties and apparently low toxicity compared to other inorganic nanoparticles, and secondly, due to their excellent colloidal stability, facile manipulation, and their nontoxic and easy synthesis methods [1,2]. Among the different types of AuNPs, the sphere-shaped AuNPs have been the most studied both for toxicological research and biomedical applications, while star-shaped AuNPs that present superior plasmonic properties and therefore a higher potential for the use in biomedical applications, have been comparatively less well studied [3,4]. Besides shape, other parameters such as size and capping are also considered important not necessarily for the field of application or plasmonic properties, but mainly due to their effect on in vivo biodistribution, clearance, and toxicity [5,6].

The vast majority of the in vitro and in vivo studies regarding the toxicity of AuNPs focus on their main target organ, the liver, and only few take into consideration other organs such as the spleen, lungs, or intestine. Among these studies, several have associated the exposure to AuNPs with toxicity and reported increased reactive oxygen species production, mitochondrial and lysosomal damage, inflammation, autophagy, DNA damage, and even apoptosis or necrosis [7,8]. These studies raise concern regarding the effect of AuNPs on other organs when they are intended for biomedical applications, since they are not biodistributed only to the liver, but can reach other organs, including the kidney [6,9,10]. In fact, the kidney with its proximal tubules and prominent role in excretion is highly susceptible to the effect of xenobiotics [11,12]. Therefore, there is an urgent need for assessing the AuNPs’ potential renal toxicity, as their interaction with the kidney can occur naturally in vivo due to their renal biodistribution and elimination. The few AuNPs nephrotoxicity studies that have been published focused on a reduced number of samples, usually just two different sized nanospheres with the same capping, that are normally not preferred for biomedical applications (i.e., ultrasmall nanoparticles or particles larger than 100 nm) [13,14].

In the present study, the in vitro toxicity of AuNPs with different shape (spheres and stars), capping (citrate and MUA) and diameters (13 and 60 nm) was investigated using an in vitro model of proximal tubular cells, the HK-2 cells, in order to elucidate their potential detrimental effect on kidney cells and the mechanisms involved.

## 2. Materials and Methods

### 2.1. Reagents

The chemicals used in the current study were of analytical grade or high purity. All reagents were purchased from Sigma-Aldrich (Lisbon, Portugal) or for the ones used in the cell culture from Gibco (Alfagene, Lisbon, Portugal). For all the other reagents, their origin was mentioned in their first appearance in the text.

### 2.2. Synthesis and Physical-Chemical Characterization of the Synthesized AuNPs

For the synthesis of 13 nm sphere-shaped AuNPs, the Turkevich–Frens method was used [15,16]. For the larger 60 nm nanospheres, the selected method was the one developed by Bastus et al. (2011) that is based on a seed-mediated process [17]. The seed-mediated growth method uses sodium citrate as a reducing agent and allows the process to be stopped when a desired size is reached [17]. For comparison purposes, half of the batch of each citrate-capped gold nanospheres were further functionalized with 11-mercaptoundecanoic acid (MUA 10 mM in ethanol) overnight, under vigorous stirring, followed by three centrifugation steps (SIGMA 3–30 K, 2000 g, 10 min, 25 °C) and redispersion in a phosphate buffer pH 7.2 solution. Star-shaped AuNPs were synthesized using the method of Yuan et al. (2011) [18]. In order to increase their stability, the obtained nanostars were further functionalized immediately after the synthesis, with MUA 10 mM in ethanol under vigorous stirring, followed by three centrifugation steps (SIGMA 3–30 K, 2000 g, 10 min, 25 °C) and redispersion in a phosphate buffer pH 7.2 solution. The colloidal suspensions were fully characterized in their suspension buffer (2.2 mM citrate solution for citrate-capped AuNPs and 10 mM phosphate buffer pH 7.4 for MUA capped-AuNPs), as previously described [19]. The characterization techniques included transmission electron microscopy (TEM), UV-Vis spectrophotometry, and graphite furnace atomic absorption spectrometry (GFAAS).

All stock suspensions were protected from light and kept at 4 °C. No sign of aggregation/precipitation was detected through visual inspection and UV/Vis analysis throughout the study. For all tested AuNPs, the same batches were used for all experiments. For all tested concentrations, the stock suspensions were firstly sonicated for 10 min in an ultrasound bath (Bandelin Sonorex RK 100H; Berlin, Germany) followed by dilution with the cell culture.

### 2.3. Cell Culture

The HK-2 proximal tubule cells, derived from normal adult human kidney, were supplied by American Type Collection (ATCC CLR-2190). The cells were routinely cultured using RPMI-1640 culture media, supplemented with 10% foetal bovine serum (FBS) and 1% penicillin-streptomycin solution in 75 cm^2^ flasks from Corning^®^ (VWR, Lisbon, Portugal). The cells were maintained in a humidified 5% CO_2_–95% air atmosphere at 37 °C, with the medium changed every 2 days. The cell passage was done when cells were below 70% confluence, using a 0.25% trypsin/EDTA solution. All experiments were performed using HK-2 cells up to a maximum of seven passages (P7–P14).

### 2.4. Cytotoxicity Assays

The cytotoxicity of AuNPs on HK-2 cells was evaluated using two viability assays, the 3-(4,5-dimethylthiazol-2-yl)-2,5-diphenyltetrazolium bromide (MTT) reduction and the neutral red (NR) incorporation assays. For that purpose, the cells were seeded onto 96-well plates at a density of 10000 cells/well, and 24 h later incubated with the nanoparticles in 10% FBS supplemented media at concentrations ranging from 1 μM to 60 μM (in Au) corresponding to concentrations between 0.06 to 3.69 μg Au/surface area cell culture dish. All samples were tested in triplicate, in at least three independent experiments. Positive, negative and solvent controls were used. The positive control corresponded to 1% Triton X-100, the negative control to cell culture media, and for the solvent controls, 2.2 mM sodium citrate solution and 10 mM phosphate buffer (pH 7.2) containing 33 μM MUA, were diluted in media to a concentration corresponding to that of the highest AuNPs concentration tested (0.2 mM sodium citrate and 1 mM phosphate buffer, respectively).

The MTT colorimetric assay was performed as previously described [19]. The method is based on the capacity of enzymes from the viable cells to reduce the MTT salt to formazan. Therefore, the cell viability and more specifically, the mitochondrial enzymes’ activity are directly proportional to the amount of obtained formazan. Briefly, after the 24 h incubation with AuNPs, the HK-2 cells were incubated with a 0.5 mg/mL MTT solution in a humidified 5% CO_2_–95% air atmosphere at 37 °C and protected from light. Two hours later, the supernatant containing the MTT solution was removed, followed by the addition of dimethylsulfoxide (DMSO, Merck, Germany) to dissolve the intracellular formazan crystals. The absorbance was measured in a multi-well plate reader (BioTek Instruments, VT, USA) at 550 nm.

The NR colorimetric assay was performed also using a previously described protocol [19]. The method relies on the incorporation and binding of NR inside lysosomes when cells are viable, while the same does not happen when cells are dead. Thus, the amount of the internalized dye can be taken as a measure of lysosomal integrity and/or cell viability. Briefly, after the 24 h exposure to AuNPs, the HK-2 cells were incubated with a 50 μg/mL NR solution in a humidified 5% CO_2_–95% air atmosphere at 37 °C and protected from light. Two hours later, the supernatant containing the NR solution was removed, followed by the addition of a lysis solution (50 ethanol: 1 glacial acetic acid: 49 water) to release and dissolve the intracellular dye. The absorbance of the coloured solution was read using a multi-well plate reader (BioTek Instruments, VT, USA) at 540 nm.

The obtained data were normalised to both negative and positive controls, and graphically presented as percentage of cell viability relative to the negative control.

### 2.5. Measurement of Reactive Oxygen and Nitrogen (ROS and RNS) Species

To evaluate the ROS and RNS production induced by AuNPs, a fluorescent assay was performed as previously described [19]. The method is based on the ability of a nonfluorescent compound, 2′,7′-dichlorofluorescin diacetate (DCFH-DA) to pass through cell membranes and reach the cytoplasm where esterase removes the acetate, producing 2’,7’-dichlorodihydrofluorescein (DCFH), which is not cell permeable because of its polarity. In the presence of ROS/RNS, DCFH can be easily oxidised into a highly fluorescent compound (2’,7’-dichlorofluorescein (DCF)). Therefore, the amount of resulting DCF can be taken as a measure of ROS/RNS production. Briefly, the HK-2 cells seeded onto 96-well plates at a density of 10,000 cells/well were firstly incubated with a 50 μM DCFH-DA probe in a humidified 5% CO_2_–95% air atmosphere at 37 °C and protected from light. Solvent controls corresponding to 0.2 mM sodium citrate solution and 1 mM phosphate buffer (pH 7.2) with 3.3 μM MUA diluted in media were also tested. After 30 min, the supernatant was eliminated. The cells were rinsed with Hank’s Balance Salt Solution (supplemented with Ca^2+^ and Mg^2+^) and incubated with AuNPs at six different concentrations ranging from 1 μM to 60 μM (0.06 to 3.69 μg Au/surface area cell culture dish), at 37 °C during 24 h. The resulting fluorescence was recorded on a fluorescence microplate reader (Power-WaveX; Bio-Tek, Winooski, VT, USA) set to 485 nm excitation and 530 nm emission. Each concentration was tested in three replicates within each experiment and results are presented as fold increase over negative control (cells incubated with the probe but without AuNPs).

### 2.6. Evaluation of Mitochondrial Integrity

To evaluate the mitochondrial depolarization, a fluorescent method was used and performed as previously described [19]. The method is based on the ability of tetramethylrhodamine ethyl ester (TMRE), a positively-charged red-orange dye, to accumulate in active mitochondria due to their relative negative charge. If the mitochondria are depolarized or inactive, the membrane potential decreases and consequently the mitochondria fail to sequester TMRE. Briefly, after the 24 h incubation with AuNPs (concentrations 1 μM to 60 μM or 0.06 to 3.69 μg Au/surface area cell culture dish), negative control (media) or solvent control (0.2 mM sodium citrate solution and 1 mM phosphate buffer (pH 7.2) with 3.3 μM MUA diluted in media), the HK-2 cells seeded at 10,000 cells/well, were incubated with a 4 μM TMRE solution in a humidified 5% CO2–95% air atmosphere at 37 °C and protected from light. Thirty minutes later, the supernatant containing the TMRE solution was removed, and cells rinsed HBSS with Ca^2+^ and Mg^2+^. The fluorescence was recorded on a fluorescence microplate reader (Power-WaveX; Bio-Tek, Winooski, VT, USA) set to 544 nm excitation and 590 nm emission. Each concentration was tested in three replicates within each experiment and results presented as the percentage of negative control (cells incubated without AuNPs).

### 2.7. Measurement of Intracellular Adenosine Triphosphate (ATP), Total Glutathione (GSHt) and of Protein Content

Quantification of ATP and total glutathione (GSHt) was performed on HK-2 cells seeded at a density of 300000 cells/well on 6-well plates. The cells were firstly incubated with 20 or 40 μM AuNPs (corresponding to 0.41 and 0.82 μg Au/surface area cell culture dish) and, 24 h later, washed twice with HBSS followed by the addition of 400 μL of 5% perchloric acid (HClO_4_, *w/v*). The cells were collected by rubber scrapping into centrifuge tubes and centrifugated at 13000 rpm, at 4 °C, for 10 min. The resulting supernatants were collected and stored at −80 °C for ATP and GSHt quantification, while the pellets were dispersed using 0.3 M NaOH for protein quantification by the method of Lawry [20].

For both ATP and GSHt determinations, 400 µL of the supernatant, of blanks (only 5% HClO_4_) and standards (0–16 μM ATP in 5% HClO_4_ or 0–15 μM GSH in 5% HClO_4_) were neutralized with the same volume of 0.76 mM KHCO_3_, on ice, and vortex-mixed to release the resulting CO_2_ from the chemical reaction. After centrifugation at 13,000 rpm, for 10 min at 4 °C, the supernatant was collected and used for ATP or GSHt determination.

The quantification of the intracellular levels of ATP in the neutralized supernatant was performed as previously described [21]. Briefly, 75 µL of these neutralized supernatants including those of test samples, blanks, and standards were transferred in triplicates into a 96-well plate and, just before reading, 75 µL luciferin–luciferase reagent [0.15 mM luciferin; 300,000 light units of luciferase from *Photinus pyralis* (American firefly); 50 mM glycine; 10 mM MgSO_4_; 1 mM Tris; 0.55 mM EDTA; 1% BSA; pH 7.6; 4 °C; protected from light] were added. The resulting luminescence was recorded using a multi-well plate reader (Power Wave XTM, BioTek Instruments, Inc), set at 560 nm emission.

The quantification of GSHt in the neutralized supernatant was performed as previously described [21]. Briefly, 100 µL of the neutralized supernatant of the test samples, blank or standards were transferred in triplicates into a 96-well plate, followed by the addition of 65 μL of fresh reagent solution containing 1.3 mM 5,5-dithio-bis(2-nitrobenzoic acid) (DTNB) and 0.24 mM *β*-NADPH in phosphate buffer (71.5 mM Na_2_HPO_4_, 71.5 mM NaH_2_PO_4_ and 0.63 mM EDTA, pH 7.5) and left to incubate in the microplate reader (Power Wave XTM, BioTek Instruments, Inc.), at 30 °C, for 15 min. Immediately before reading, 40 μL of glutathione reductase solution (10 U/mL) were added and the formation of 5-thio-2-nitrobenzoic acid (TNB) was recorded for 3 min using a multi-plate reader (PowerWaveX; Bio-Tek Instruments, Winooski, VT, USA) at 415 nm. The GSHt concentrations of the test samples were extrapolated using the standard curve.

For the protein quantification, the pellets dispersed in 0.3 M NaOH were diluted using the same solvent and 50 μL of these samples, of BSA standard, and blank (only 0.3 M NaOH) were transferred in triplicates into a 96-well plate. Afterwards, 100 μL of reagent A (14.7 mL of 2% Na_2_CO_3_, 0.15 mL of 2% KNaC_4_H_4_O_6_.4H_2_O, and 0.15 mL of 1% CuSO_4_.5H_2_O) were added to each well and left to incubate at room temperature, protected from light. Ten minutes later, 100 μL of reagent B (Folin and Ciocalteu’s phenol reagent, diluted 1:15 with distilled water) were added and left to incubate under the same conditions. Twenty minutes later, the absorbance was recorded using a multi-well plate reader (BioTek Instruments, VT, USA) at 750 nm. Each sample, blank or standard was tested in triplicate. Results were extrapolated from the calibration curve.

Result for ATP and GSHt determination were normalized to total protein content and presented as nmol/mg protein. Solvent controls corresponding to 0.2 mM sodium citrate solution and 1 mM phosphate buffer (pH 7.2) with 3.3 μM MUA diluted in media were also tested.

### 2.8. Caspase 3, 8 and 9 Determination

The colorimetric determination of caspase 3, 8 and 9 activity in HK-2 cells was performed as previously described [22]. The method is based on the release of the *p*-nitroanilide moiety from the substrate (Ac-DEVD-pNA for caspase 3, Ac-IETDpNA for caspase 8 and Ac-LEHD-*p*NA for caspase 9) in the presence of caspase (3, 8 and 9, respectively). Therefore, the amount of resulting *p*-nitroanilide moiety can be related to caspase activity. Briefly, the HK-2 cells seeded onto 6-well plates at a density of 300,000 cells/well were firstly incubated with 20 or 40 μM AuNPs (corresponding to 0.41 and 0.82 μg Au/surface area cell culture dish) and, 24 h later, the cells were washed twice with HBSS followed by the addition of 150 μL lysis buffer (50 mM HEPES, 0.1 mM EDTA, 0.1% CHAPS, supplemented with 1 mM DTT, pH 7.4). The cells were collected by rubber scrapping and transferred into centrifuged tubes, vortex-mixed, incubated on ice (5 min), and centrifugated (13,000 rpm, 10 min, 4 °C). Then, 50 μL of the obtaining supernatant (cytoplasmic fraction) were transferred into a 96-well plate. To each well was added 200 μL of assay buffer (100 mM NaCl, 50 mM HEPES, 1 mM EDTA, 0.1% CHAPS, 10% glycerol, supplemented with 10 mM DTT, pH 7.4). For the reaction to start, 5 μL of peptide substrates were added to each well. The peptide substrate was caspase-3 (Ac-DEVD-*p*NA, 4 mM in DMSO), caspase-8 (Ac-IETDpNA; 10 mM in DMSO) or caspase-9 (Ac-LEHD-pNA; 10 mM in DMSO). The plates were further incubated for 24 h at 37 °C, protected from light. The resulting absorbances were recorded on a multi-well plate reader (Power Wave XTM, BioTek Instruments, Inc.) at 405 nm. The results were calculated by subtracting the absorbance of the blanks (non-enzymatic control) from each absorbance value of the test samples and further normalized to the amount of protein of each sample.

In order to quantify the protein content, the Bio-Rad DC protein assay kit was used as suggested by the manufacturer (Bio-Rad Laboratories, Amadora, PT). A calibration curve of bovine serum albumin (BSA) was used with standard solutions ranging from 31.25 μg/mL to 1000 μg/mL.

The caspase results are presented as percentage of negative controls from three independent experiments. Solvent controls corresponding to 0.2 mM sodium citrate solution and 1 mM phosphate buffer (pH 7.2) with 3.3 μM MUA diluted in media were also tested and graphically presented as a percentage of negative control.

In order to verify the lack of any absorbance or interference in the experimental settings, any possible interference of the AuNPs was taken into consideration and all our experiments always included controls without cells but containing each of the tested gold nanoparticles concentration.

### 2.9. Statistical Analysis

The statistical analysis was performed using the GraphPad Prism 6 software (GraphPad Software, San Diego, CA, USA). For the MTT and NR assays, the results were presented as mean ± standard error of the mean (SEM) from four independent experiments run in triplicates. Normality of the data distribution was assessed by the Kolmogorov–Smirnov, D’Agostino & Pearson and Shapiro–Wilk tests. The solvent and negative controls were statistically compared using the un-paired Student t-test, in all data. If a statistically significant difference would be found between negative control and solvent, the final statistical analysis would be done by comparing the test concentration to the corresponding solvent control and not the negative control, as normally done. For the groups statistical comparisons, one-way analysis of variance (ANOVA) followed by the Tukey’s multiple comparison test were applied. In case of data without normal distribution, the Kruskal–Wallis test followed by the Dunn’s post hoc test were used. Significance was considered when *p* values ≤ 0.05.

## 3. Results

### 3.1. Synthesis of Citrate- and MUA-Coated Nanospheres of 13 and 60 nm and MUA-Coated Gold Nanostars of 60 nm

**Figure 1:**
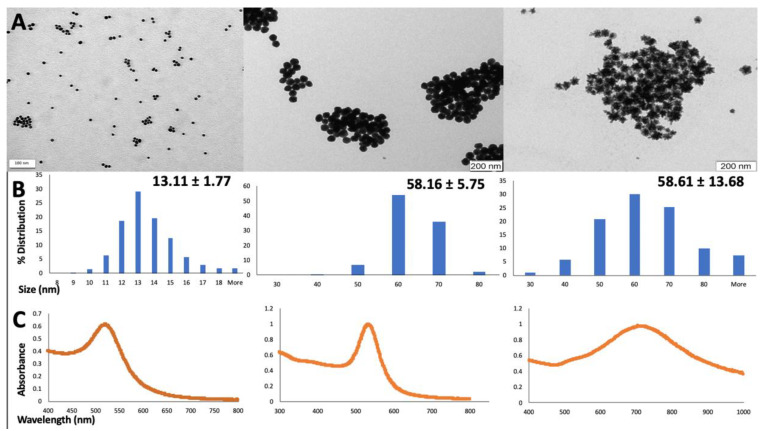
Physical-chemical characterization of the 13 and 60 nm gold nanospheres and 60 nm gold nanostars. Data obtained from (**A**) TEM analysis, (**B**) size distribution by frequency obtained from TEM images and (**C**) their corresponding UV-Vis spectra. TEM images were analyzed using ImageJ software through measurement of a minimum of 100 nanoparticles. For the UV-Vis spectra, the samples were diluted 1 to 5 in 2.2 mM citrate solution for citrate-capped AuNPs and 10 mM phosphate buffer pH 7.2 for MUA capped-AuNPs.

Gold nanospheres with two distinct sizes (13 and 60 nm) and coated with citrate or MUA and MUA-coated gold nanostars were successfully synthesized and characterized using UV-Vis spectroscopy and TEM analysis (Figure 1).

The TEM analysis demonstrated that the diameter of the smaller nanospheres was 13.11 ± 1.77 nm, while the larger nanospheres presented a diameter of 58.16 ± 5.75. Gold nanostars were obtained with a size diameter of 58.61 ± 13.68 nm. The UV-Vis spectra showed a maximum peak of absorption around 521 nm wavelength for the smaller 13 nm nanospheres, 535 nm wavelength for the larger 60 nm nanospheres, and 704 nm wavelength for the 60 nm nanostars (Figure 1C). No secondary peaks or artefacts were detected in any of the UV-Vis analysis. The gold mass concentrations of the stock suspensions, as determined by GFAAS analysis correspond to 114.60 ± 5 μg/mL for 13 nm citrate-AuNPs, 103.81 ± 5 μg/mL for 13 nm MUA-AuNPs, 104.83 ± 3 μg/mL for 60 nm citrate-AuNPs, 107.93 ± 1 μg/mL for 60 nm MUA-AuNPs and 147.37 ± 7 μg/mL for 60 nm MUA-nanostars.

### 3.2. All Tested AuNPs Induced a Concentration-Dependent Cytotoxicity on HK-2 Cells

**Figure 2:**
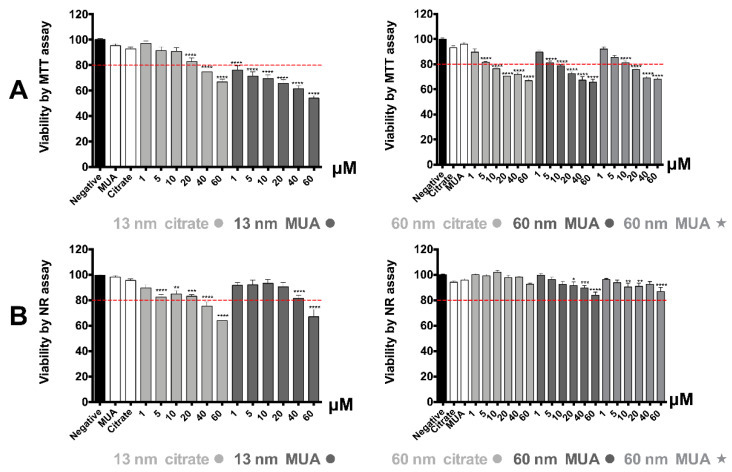
Cytotoxicity elicited by the AuNPs in HK-2 cells. Data obtained in the (**A**) 3-(4,5-dimethylthiazol-2-yl)-2,5-diphenyltetrazolium bromide (MTT) viability assay and (**B**) the neutral red incorporation assay in the lysosomes, after 24 h incubation at 37 °C. Results are from four independent experiments, performed in triplicate, and are presented as percentage of cell viability, relative to the negative controls and positive controls. Statistical comparisons were made using one-way ANOVA/Kruskal–Wallis test. * *p* ≤ 0.05; ** *p* ≤ 0.01; *** *p* ≤ 0.001; **** *p* ≤ 0.0001, vs. control. The dotted red lines represent 80% viability.

As can be seen in Figure 2A, all tested AuNPs produced a significant concentration-dependent decrease, of more than 20%, of HK-2 cells metabolic activity versus negative control (cell culture media), as determined by the MTT reduction assay. The most toxic were the 13 nm MUA-capped nanospheres, for which the cellular metabolism was reduced up to 44% versus the negative control (*p* ≤ 0.0001). The AuNPs effect on the lysosomal integrity, as shown by the NR assay, was not as severe (Figure 2). The NR assay showed a significant reduction of cell viability of approximately 37% versus negative control, only for the 13 nm citrate and MUA-capped nanospheres. The effect was statistically significant for the 13 nm citrate-capped nanospheres at the highest 40 μM and 60 μM tested concentrations while for the 13 nm MUA-capped nanospheres only at 60 μM (*p* ≤ 0.0001) (Figure 2B). MTT was therefore more sensitive than the NR assay for the evaluation of cytotoxicity effect of the AuNPs.

### 3.3. AuNPs Effects Are Associated to a Decrease in Mitochondrial Membrane Potential and an Increase in ROS/RNS Production at the Highest Tested Concentration

**Figure 3:**
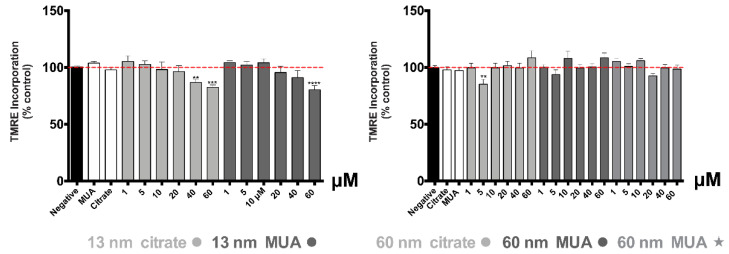
Mitochondrial membrane potential (Δ*ψ*m) indirectly assessed by the TMRE incorporation in mitochondria of HK-2 cells, after 24 h incubations at 37 °C with AuNPs. Results from at least four independent experiments, run in triplicates, are expressed as percentage control ± standard error of the mean (SEM). Statistical comparisons were made using one-way ANOVA/Dunnett’s post hoc test ** *p* ≤ 0.01; *** *p* ≤ 0.001; **** *p* ≤ 0.0001 versus control.

A decrease in the mitochondrial membrane potential, as assessed by the TMRE assay, was found for both the 13 nm citrate- and MUA-capped nanospheres, but not for the larger 60 nm nanospheres and nanostars (Figure 3). The membrane depolarization occurred at the highest tested concentration (40 and 60 μM for the citrate-AuNPs of 13 nm and 60 μM for their MUA-counterparts) (*p* ≤ 0.01).

**Figure 4:**
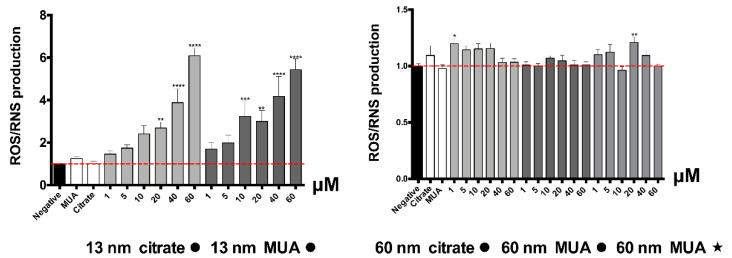
Intracellular reactive species, as assessed by the oxidation of the DCFH dye, in HK-2 cells, after 24 h incubations at 37 °C with 1–60 μM of AuNPs. Results from at least four independent experiments, run in three replicates, are expressed as fold increase over control. Statistical comparisons were made using one-way ANOVA/Kruskal–Wallis test. * *p* ≤ 0.05; ** *p* ≤ 0.01; *** *p* ≤ 0.001; **** *p* ≤ 0.0001, vs. control.

The toxicological effect of the 13 nm nanospheres was associated to a large increase of ROS/RNS production (Figure 4). The effect was statistically significant starting from 10 μM for the MUA-capped nanospheres (*p* ≤ 0.001), and from 20 μM for their citrate counterparts (*p* ≤ 0.01).

### 3.4. The Energetic Status and Antioxidant Defence Was only Altered in HK-2 Cells Exposed to 13 nm MUA-AuNPs

**Figure 5:**
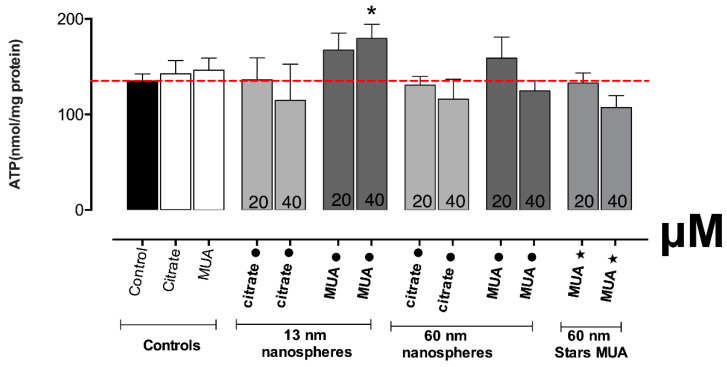
Intracellular ATP levels, in HK-2 cells, after 24 h incubations at 37 °C with AuNPs. Results from three independent experiments are expressed as mean ± standard error of the mean (SEM). Statistical comparisons were made using Kruskal–Wallis one-way analysis of variance/Dunnett’s post hoc test. * *p* ≤ 0.05; versus control.

The mitochondrial damage observed for the 13 nm MUA-capped nanospheres was reflected also in the results obtained for the ATP assay (Figure 5). The exposure to the concentration of 20 μM leads to a slight increase in ATP production, and for the 40 μM, this effect increased further, reaching statistical significance (*p* ≤ 0.05). None of the other tested AuNPs produced altered ATP levels on HK-2 cells.

**Figure 6:**
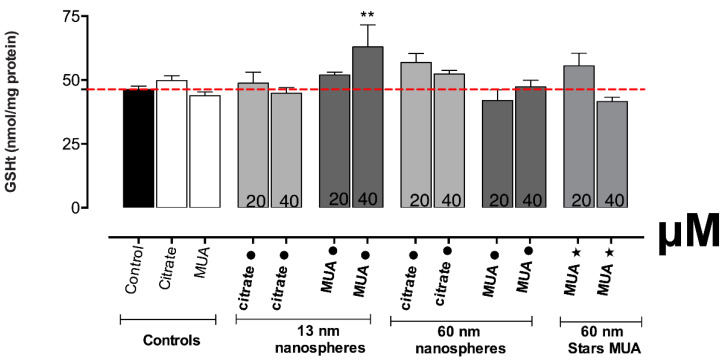
Intracellular contents of total glutathione (GSHt), in HK-2 cells, after 24 h incubations at 37 °C with AuNPs. Results from four independent experiments are expressed as mean ± standard error of the mean (SEM). Statistical comparisons were made using one-way ANOVA/Dunnett’s post hoc test ** *p* ≤ 0.01; versus control.

In accordance with the increase in ROS/RNS production for the 13 nm MUA-capped nanospheres, also the antioxidant defense as assessed through GSHt determination, was found significantly increased at the 40 μM (*p* ≤ 0.01) and slightly increased at the 20 μM tested concentration (Figure 6). The same results were not confirmed for the citrate-capped counterparts or all the other tested AuNPs, where no significant differences were found for any of the tested concentrations versus negative control.

### 3.5. The Smaller 13 nm MUA-AuNPs Activated Both Extrinsic and Intrinsic Markers of Apoptosis

**Figure 7:**
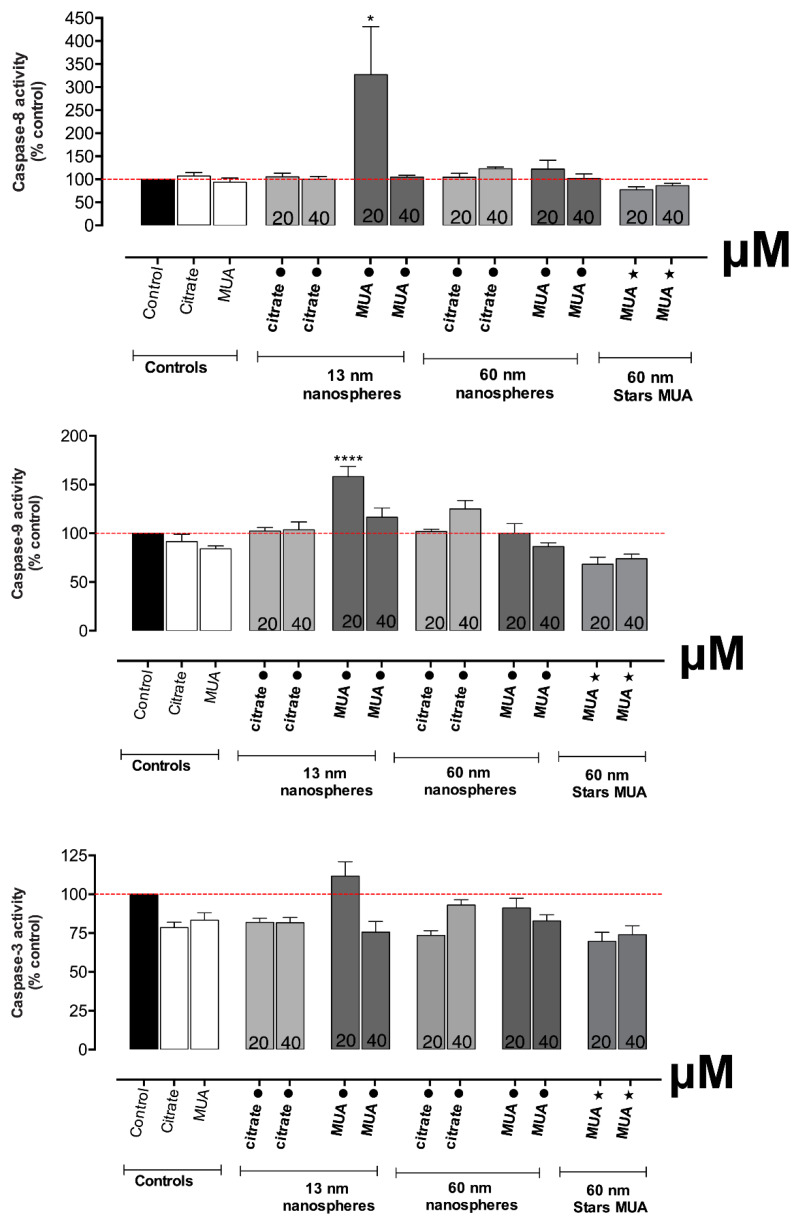
Caspase-8, -9, and -3 activity in HK-2 cells, after 24 h-incubations at 37 °C with AuNPs. Results from three independent experiments are expressed as percentage control ± standard error of the mean (SEM). Statistical comparisons were made using sing one-way ANOVA/Dunnett’s post. * *p* ≤ 0.05, **** *p* ≤ 0.0001 versus control.

As can be seen in Figure 7, the 13 nm MUA-capped nanospheres, at 20 μM tested concentration, significantly activated the caspase 8 activity, an extrinsic factor of apoptosis (*p* ≤ 0.05). The same was noticed for the caspase-9 activity (*p* ≤ 0.0001) associated to a slight increase of the caspase-3 activity, but without reaching statistical significance. No other tested AuNPs demonstrated pro-apoptotic effects.

## 4. Discussion

The HK-2 is a human immortalized cell line used to predict the nephrotoxicity of various xenobiotics including inorganic nanoparticles, especially on tubular proximal cells [14,23,24]. Its ability to elucidate the renal toxicity mechanisms of xenobiotics is based on the cells’ expression of many enzymes and transport proteins that mediate the filtration and reabsorption in the kidneys [23]. Nonetheless, this cell model presents some limitations as their transporter expression has not yet been fully characterized and it lacks the in vivo complexity [23,25]. Despite these limitations, the HK-2 cells are considered a good alternative for primary cells.

Concerns regarding AuNPs renal effect were raised since their in vivo biodistribution and clearance can affect also the kidneys and can easily penetrate into renal cells [26,27,28]. Several studies indicate that the smaller the size of the AuNPs, the higher the probability of being eliminated mainly through the kidney versus the hepatobiliary system [26,27]. In fact, AuNPs larger than 6 nm seem to be unable to undergo glomerular filtration, a critical step in urinary clearance. For example, Yang et al. (2014) demonstrated that CD1 mice injected intravenously with 4.5 nm PEG-coated nanospheres presented after 5 h a higher Au content in kidney and later in urine (24 h), compared to 30 nm counterparts [27]. In spite of the concern regarding AuNPs’ biocompatibility, the information regarding their toxicological effect on kidneys and its dependence on their physical-chemical parameters as the size, is still scarce [13,14].

The AuNPs synthesis and characterization methods were identical to those used in previously published studies of our group [19,21,29]. As always, these methods were selected to produce nanoparticles with specific shapes (spheres and stars), high reproducibility of size and shape and narrow size dispersion as these are considered important parameters for the AuNPs toxicological investigation [17,18]. In order to assess the physical-chemical parameters of the synthesized AuNPs and their colloidal stability, some of the widely used techniques including UV-Vis spectroscopy and TEM analysis were performed [30,31]. While TEM analysis gives accurate information regarding the shape and size of the AuNPs, the UV-Vis technique can be successfully used both for confirming the results obtained from previous TEM analysis and also to inform about the AuNPs aggregation state and capping. This is possible as AuNPs plasmon resonances gives specific spectral signatures dependent firstly on the type of nanoparticles but also on their characteristics including size, shape, capping agent and aggregation state. The plasmonic bands obtained for the 13 nm gold nanospheres, 60 nm gold nanospheres and 60 nm gold nanostars are consistent with previous studies of our group [29] and also with other reports from the literature [8,32].

Some of the most common viability assays used for the study of AuNPs cytotoxicity are the MTT and NR assays [7,33,34] as they do not present any risk of interference with the optical properties of the AuNPs [35]. All tested AuNPs demonstrated more severe toxicity through MTT assay compared to NR. This outcome suggests that AuNP’s effect is preferentially oriented towards cellular metabolic activity, and particularly to mitochondrial metabolism, and to a lower extent towards other cellular organelles as lysosomes. This is in accordance with a previous study of our group that demonstrated that AuNPs with similar physical-chemical properties to the ones in this study demonstrated higher toxicity at the same tested concentrations and incubation time with the MTT assay as compared to the NR [19]. The higher toxicity towards mitochondria would be expected as the literature indicates that mitochondria is the main target organelle of AuNPs’ toxicity [7,14]. Both MTT and NR assays demonstrated a more severe toxicity for the 13 nm nanospheres as compared with the larger 60 nm ones, consistent with previously published trends [14,36]. This is very important if we consider that small AuNPs have a higher ability to reach the kidney cells after systemic administration [27]. Nevertheless, as extensively discussed in a previously published paper of our group, the fact that HK-2 cells were exposed to AuNPs with the same concentration in Au mass, does not correspond to the same number of nanoparticles available for cellular uptake and reaching the mitochondria [19] so caution should be taken when a direct comparison is made.

Normally, severe damage of mitochondrial function is associated with significant ROS induction and changes in the redox state (as reflected by the intracellular glutathione levels). In fact, increased levels of intracellular ROS were found for the 13 nm nanospheres in our study. The increase in ROS/RNS production is considered a critical indicator of the oxidative stress described as a major factor of AuNPs’ toxicity [37]. Increased levels of total glutathione were also found, even if only for the 13 nm MUA-capped nanospheres, contributing to the hypothesis of a possible disturbance in GSH levels that can be related to oxidative stress events. It is known that in case of oxidative stress, GSH is crucial for neutralization of toxic metabolites/substances by forming GSH adducts [38] or through direct reactivity producing oxidized glutathione [39,40]. The increase found for 13 nm MUA-capped nanospheres could therefore be explained by a compensatory mechanism for the replacement of consumed GSH with a newly synthesized one [38,41]. The differences in the oxidative stress and disturbed GSH levels could be due to different levels of toxicity, with MUA capping presenting higher toxicity than citrate. The severe mitochondrial damage produced especially by the 13 nm nanospheres is confirmed also by the significant disruption of the mitochondrial membrane potential.

The exposure to 13 nm MUA-capped AuNPs activated also cellular apoptosis as shown by the increase in caspase 8 and 9 activities, an important process for maintaining the cellular homeostasis in terms of cell division and cell death. This type of cellular death could be a synergetic effect of mitochondrial disfunction as the loss of mitochondrial membrane potential is known to be capable of enhancing or inhibiting several key regulators of apoptosis [14]. Other AuNPs, such as 5 nm gold nanospheres (50 nM), were already reported to induce apoptosis associated with an increase in ROS production and mitochondrial membrane depolarization in hypoxic HK-2 cells, 24 h after incubation [14]. The fact that caspase activation is noticed only at 20 µM and not at 40 µM suggests the involvement of other processes besides apoptosis, especially at the higher tested concentrations, such as autophagy, that has already been associated to the AuNPs effects [14].

The fact that the larger 60 nm nanospheres and nanostars only altered metabolic activity as shown by the MTT assay, without affecting the mitochondrial membrane potential, lysosomal integrity, ROS/RNS production, energetic and redox status or activating apoptosis, suggests that these AuNPs could manifest only an early-phase cytotoxicity without affecting the normal functioning of the cells. In accordance with the low toxicity towards kidney cells found in our study for the larger 60 nm gold nanospheres, Ibrahim et al. (2018) did not detect any toxic effect on the kidneys of the Swiss albino mice, 24 h after intraperitoneal injection of 50 nm hexagonal-shaped AuNPs at a dose of 170 μg/kg bodyweight [36]. The slight histopathologic changes that were found in the kidneys including diminished and distorted glomeruli, did not reach significance as compared with controls. Even more, biomarkers of oxidative stress such as glutathione and malondialdehyde were found unaltered [36]. Nevertheless, this does not suggest that all AuNPs of similar size would manifest a low toxicity to kidney cells. For example, Khan et al. (2013) found that 50 nm AuNPs of hexagonal shape injected intraperitoneally to Wistar-Kyoto rats (22 μg/kg bodyweight), induced an acute phase increase in proinflammatory cytokines expression including IL-6 and TNF-*α*, 24 h after administration [42]. Therefore, each nanoparticle should be tested individually as many other factors including the biological model tested, shape, capping, concentration, and exposure time are known to influence the outcome.

Regarding the 13 nm AuNPs, especially the MUA-capped ones, the manifested toxicity could reach an intermediate-phase when the cells are forced to activate their defence to neutralize the toxic process. In accordance to our results for the 13 nm nanospheres, Tlotleng et al. (2016) found by using label-free real-time cell analyzer impedance technology, that 13 nm citrate- and PEG-capped gold nanospheres induce a significant decrease in the cell viability of the human embryonic kidney (HEK 293) cells, 24 h after incubation with concentrations between 0.5–5.0 nM [43]. Nevertheless, not all published studies are in agreement. For example, Ding et al. (2014) found no changes in the cellular viability of HK-2 cells, both by the MTT and the LDH assays, 24 h after incubation with 13 nm citrate-capped gold nanospheres, at concentrations between 0–50 nM [14]. These differences could be explained by the use of different assays, concentrations and synthesis method.

The high toxicity noticed for the 13 nm MUA-capped nanospheres could be explained through its size and capping, parameters known to influence the toxicity and cellular uptake [44,45,46]. It could be hypothesized that MUA-capped AuNPs are internalized to a greater extent than the citrate-capped ones that lead to a higher toxicity for 13 nm MUA-AuNPs compared to citrate ones. An uptake-dependent toxicity was found also in previous studies of our group. In those cases, the citrate-capped AuNPs of similar diameters (14–15 nm) presented higher cellular uptake than MUA-capped ones in hCMEC/D3 and Caco-2 cell lines that were associated to higher toxicity [19,29]. Similarly, other authors found a capping-dependent toxicity related to the capping-dependent cellular uptake [47,48]. The differences regarding citrate versus MUA-comparison can appear, as different cellular models were used that have already been described to influence the AuNPs uptake [14]. In the same way could be explained the higher toxicity for 13 nm nanospheres compared to larger 60 nm counterparts, as size is another critical factor for AuNPs toxicity and uptake [14,49].

Additionally, it could be hypothesized that MUA-capped AuNPs are internalized mainly through a distinct pathway compared to the citrate-capped ones and have a distinct intracellular faith that contributes to its severe toxicity. In fact, Guarnieri et al. (2014) suggested that the nanotoxicity of metallic nanoparticle and their intracellular accumulation inside the endolysosomal compartment is directly related to the nanotoxicity [50]. The AuNPs uptake occurs mainly through receptor-mediated endocytosis including clathrin- and caveolin-mediated endocytosis, and usually one predominates over the other, dictating the fate of the AuNPs inside the cellular compartment [44,49,51]. The clathrin-mediated uptake normally leads to a sequestration inside lysosomes and further exocytosis of the internalized nanoparticles, while the caveolae-mediated endocytosis avoids the sequestration and lysosomal destruction allowing therefore more interaction between the nanoparticles and cellular components with a consequently possible higher toxicity [52,53]. It could be hypothesized that for the 13 nm MUA-capped AuNPs, the internalization is done in HK-2 cells preferentially through a caveolae-mediated process, and this could explain the higher toxicity. Totleng et al. (2014) demonstrated that 14 nm citrate-capped nanospheres are localized mainly free in the cytoplasm in HEK-293 cells at the end of 24 h incubation, while PEG-capped nanospheres were localized both in the cytoplasm and large vesicles, confirming that different capping can lead to different intracellular localization [43]. For HK-2 cells, Ding et al. (2014) found that 5 nm spherical nanoparticles internalize after 24 h, as small or large aggregates mainly into lysosomes [45], but no comparison was done with other type of AuNPs.

## 5. Conclusions

In conclusion, the in vitro toxicological assessment of AuNPs with different shapes (spheres and stars), capping (citrate and MUA), and diameters (13 nm and 60 nm) demonstrated that, for this cellular model of the kidney, the type of AuNPs selected, including their size, shape and capping, are critical factors affecting toxicity. In fact, the 13 nm nanospheres proven to be most toxic to HK-2 cells, producing mitochondrial and lysosomal damage, were associated to increased ROS production. For the 13 nm MUA-capped AUNPs, the toxicity was severe, leading to changes in the energetic and redox status of the cells and activation of both extrinsic and intrinsic markers of apoptosis. On the other hand, larger 60 nm AuNPs, both nanospheres and nanostars are apparently less toxic than their smaller counterparts but cellular viability assessed by MTT assay was nevertheless significantly reduced. These findings are especially important as the size of the AuNPs proved to significantly influence the renal clearance of AuNPs.

Considering the results herein presented, concerns are raised regarding a potential detrimental effect of the AuNPs on kidney cells and later compromised kidney function, especially for the 13 nm gold nanospheres, after systemic administration. The larger 60 nm AuNPs demonstrated better biocompatibility and could be preferentially taken into consideration for systemic administration, but extensive biodistribution and in vivo toxicological studies on kidney and other organs including liver are still required.
